# Protective, Biostimulating, and Eliciting Effects of Chitosan and Its Derivatives on Crop Plants

**DOI:** 10.3390/molecules27092801

**Published:** 2022-04-28

**Authors:** Maria Stasińska-Jakubas, Barbara Hawrylak-Nowak

**Affiliations:** Department of Botany and Plant Physiology, Faculty of Environmental Biology, University of Life Sciences in Lublin, Akademicka 15, 20-950 Lublin, Poland; maria.jakubas@up.lublin.pl

**Keywords:** chitosan, biostimulants, biotic elicitor, polycationic polymers, secondary metabolites

## Abstract

Chitosan is a biodegradable and biocompatible polysaccharide obtained by partial deacetylation of chitin. This polymer has been gaining increasing popularity due to its natural origin, favorable physicochemical properties, and multidirectional bioactivity. In agriculture, the greatest hopes are raised by the possibility of using chitosan as a biostimulant, a plant protection product, an elicitor, or an agent to increase the storage stability of plant raw materials. The most important properties of chitosan include induction of plant defense mechanisms and regulation of metabolic processes. Additionally, it has antifungal, antibacterial, antiviral, and antioxidant activity. The effectiveness of chitosan interactions is determined by its origin, deacetylation degree and acetylation pattern, molecular weight, type of chemical modifications, pH, concentration, and solubility. There is a need to conduct research on alternative sources of chitosan, extraction methods, optimization of physicochemical properties, and commercial implementation of scientific progress outcomes in this field. Moreover, studies are necessary to assess the bioactivity and toxicity of chitosan nanoparticles and chitosan conjugates with other substances and to evaluate the consequences of the large-scale use thereof. This review presents the unique properties of chitosan and its derivatives that have the greatest importance for plant production and yield quality as well as the benefits and limitations of their application.

## 1. Introduction

Chitosan is a biopolymer obtained from chitin, which is the second most common natural polysaccharide after cellulose [1,2]. The discovery and first research on this compound date back to the 19th century, when the links between chemistry, botany, and medicine were discerned. Chitin was probably discovered in 1799 by English scientist A. Hachett, who extracted the compound from shells of marine invertebrates and described it as “a material with particular resistance to ordinary chemicals”. However, he did not conduct further research on this compound. For this reason, it is believed that chitin, originally called fungine, was first isolated from fungi by French researcher H. Braconnot and subsequently described by Swiss chemist A. Hoffman in his doctoral thesis. Almost 20 years later, the same compound was isolated from insect cuticle by A. Odier and named chitin (Greek: chitōn—tunic, coat). In turn, chitosan was discovered accidentally in 1859 by French physiologist C. Rouget during the production of natural soap. The process of boiling water-dissolved chitin with the addition of concentrated potassium hydroxide resulted in the deacetylation of chitin in the alkaline solution yielding chitosan. Intensive research on the structure and properties of chitin and chitosan was carried out in the following years. The modified form of chitin was eventually named “chitosan” by German chemist and physiologist F. Hoppe-Seiler in 1894; however, the chemical structure of this compound was determined only in the mid-20th century [3,4,5,6,7].

**Figure 1:**
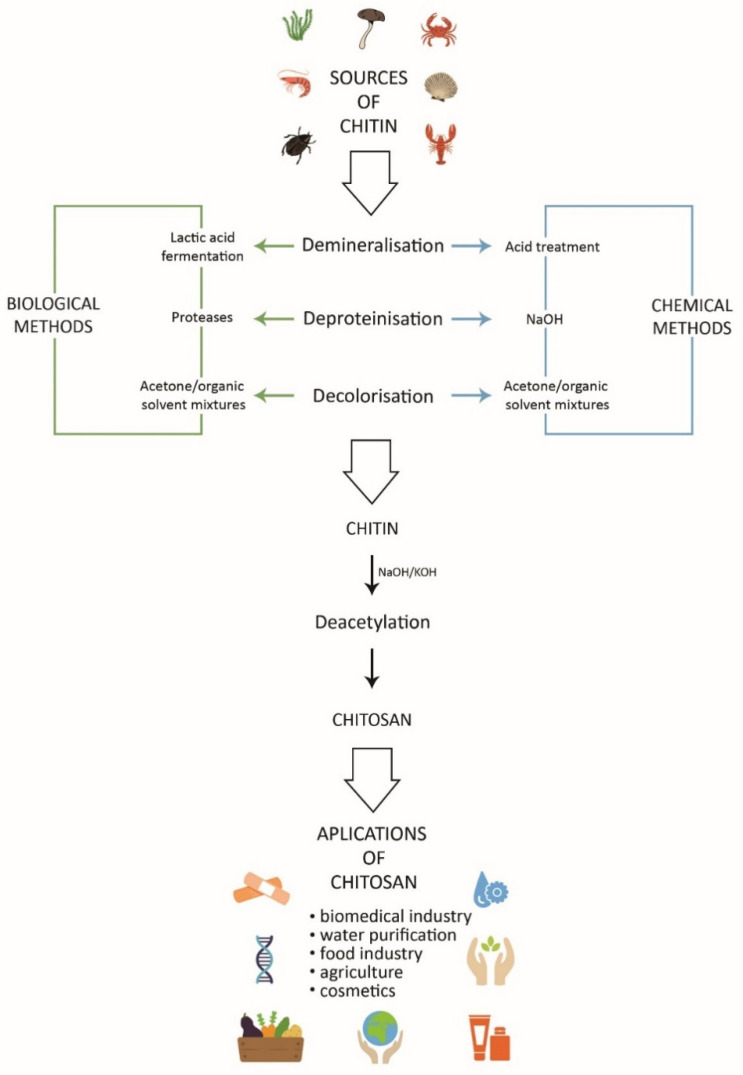
Extraction, preparation, and applications of chitosan and chitosan derivatives.

Currently, the term chitosan denotes a group of biopolymer substances obtained in the process of chemical or enzymatic deacetylation of chitin with different (but not lower than 50%) deacetylation degrees. Both chitosan and chitin are linear copolymers consisting of D-glucosamine and N-acetyl-D-glucosamine linked together by β-1,4-glycosidic bonds. The main difference between these polymers is their deacetylation degree (DD) expressed as a percentage and defined as the ratio of the number of amino groups (–NH_2_) to the total number of acetylamino groups (–NHCOCH_3_) present in chitin [1,4,8]. In addition, the solubility in dilute acids is a practical criterion for discriminating chitosan from chitin. The extraction of chitin begins with demineralization aimed at dissolving the calcium carbonate by acid treatment followed by deproteinization which uses, depending on the method (biological or chemical), enzymes or alkaline solutions. In order to obtain pure and colorless chitin, the last step is decolorization (Figure 1). The deacetylation process results in the removal of the acetyl groups (–CH_3_CO) from chitin and substitution of reactive –NH_2_ groups, thus resulting in the formation of chitosan, whose deacetylation degree depends primarily on the duration of the reaction, the temperature (90–120 °C), and the concentration of the aqueous NaOH solution (40–50%) used in the process. Although it occurs naturally in the cell walls of some fungi, bacteria, and algae as well as insect cuticles, chitosan for industrial and laboratory use is obtained from chitin derived mainly from the shells of marine invertebrates (shrimps, crabs, lobsters, and krill), which are wastes of marine food processing industry [1,8,9,10]. Until recently, the use of marine resources was considered one of the most effective methods for the extraction of chitin and the production of chitosan. An additional advantage was the possibility of disposal of a huge amount of waste generated by the marine food sector. However, some disadvantages and threats posed by this practice have been noticed in recent years, e.g., dependence on the fish industry, environmental hazards, and allergenicity of the final product, which depends on its purity. According to Ravindranathan et al. [11], chitosan thoroughly purified from impurity proteins and endotoxins is low-allergenic. However, the global chitin and chitosan market is developing very dynamically, and it has become important to look for alternative sources of these polymers for commercial and sustainable production. Currently, an increase in the number of studies on the production and applications of chitosan from various insect species can be observed. The primary cause of the interest is the speed of insect reproduction and the possibility of year-round breeding. Additionally, the results of many studies show that insect chitosan has even more favorable properties than that derived from shellfish. Recently, there have been attempts to produce chitosan from chitin derived from cell walls of fungi, which are the second-largest source of chitin after marine invertebrates [10,12,13,14,15], and biological methods are increasingly being used to obtain these polymers [2].

Chitosan is a compound with a number of physicochemical properties determining its suitability to be used in medicine, pharmacy, environmental protection, and agriculture as well as food, cosmetic, textile, and paper industries. These properties include nontoxicity; biodegradability; biocompatibility; hydrophilicity; film-forming properties; high sorption capacity; and high affinity for metals, proteins, and dyes. A summary of the extraction and applications of chitosan is presented in Figure 1. However, chitosan also exhibits many physicochemical properties that impede its wider use in some areas. The main limitation is its solubility, which largely depends on the isolation conditions, degree of deacetylation, type of acetyl group distribution, and molecular weight. There are many types of chitosan which are well soluble in both alkaline and neutral media, but in most cases, the best solvents for this group of polysaccharides are some acids. Additionally, chitosan swells strongly in an acidic environment and is characterized by poor mechanical and chemical strength. Therefore, chitosan is subjected to various types of physical and chemical modifications primarily aimed at improvement of its solubility and other physicochemical properties, e.g., mechanical strength, thus expanding the spectrum of its potential applications [8,13,16,17,18,19]. Three basic groups of chitosan modification techniques are used, i.e., methods leading to an increase in the molecular weight or extension of the chitosan chain; generation of chitosan derivatives through substitution reactions; and methods of physical, chemical, or enzymatic depolymerization. Chitosan derivatives obtained through modifications are an important object in research on their application in biotechnology, agro-technology, and medicine as well as food, pharmaceutical, and textile industries [20,21].

## 2. Biological Activity of Chitosan

Chitosan is a biopolymer with a wide potential for application in plant production mainly due to its biocompatibility and biodegradability as well as high biological activity. Although it is not part of the structure of plant tissues, chitosan exerts a considerable impact on plant growth and development. It initiates and modulates various types of physiological reactions, e.g., defense and immune responses in plants, and regulates metabolic processes [22]. Chitosan also exhibits strong antibacterial, antifungal, and antiviral properties. The first investigations of its antimicrobial activity were reported in 1979 by Allan and Hadwiger, whose publication contributed to the growing interest in the potential application of chitosan in the agricultural industry. However, the practical use of this compound in plant production became possible with the wider availability of industrial amounts of chitosan. The compound has been used for protecting plants against pathogens and fighting diseases affecting plants during the vegetation and postharvest period only since the 1990s [23].

The mechanism of the antimicrobial activity of chitosan has not been fully elucidated yet, but many literature reports indicate that it is most probably related to its polycationic nature. The interactions of chitosan molecules with negatively charged molecules on the surface of microbial cells lead to agglutination of their cell walls, loss of intracellular components, and cell death. Another hypothesis assumes that chitosan limits the entry of pathogens into plant cells by reduction in stomatal opening. In turn, the antifungal activity of chitosan results from its ability to bind and inhibit the synthesis of toxins and stimulate induced systemic resistance and production of many secondary fungistatic metabolites, e.g., phytoalexins, abscisic acid, methyl jasmonate, and phenolic compounds, and various enzymes such as hypha-degrading chitinase and β-glucanase. Additionally, microscopic studies have shown that chitosan induces clear morphological changes in fungal cells at various stages of development. This polymer also reinforces plant cell walls through lignification and constitutes a mechanical barrier limiting plant contact with adverse external factors [4,24,25,26,27,28,29]. Consequently, the effectiveness of chitosan action against microorganisms is associated with its bidirectional action: control of the presence of pathogens and induction of plant defense reactions [30].

The high biological activity of chitosan in the induction of plant immune response may be related to the processes taking place in plant cell walls. Acid pectins present in the cell walls bind calcium and form chain dimers at higher concentrations of the element. Cationic chitosan can interact with negatively charged pectin and pectin dimers, thus influencing their supramolecular conformation, which induces a specific alarm signal informing plant cells about the degradation of cell walls and the presence of pathogens. The plant response to chitosan–pectin dimer complexes is considerably stronger than that in the case of separately interacting components [31].

Chitosan has been shown to have antioxidant activity consisting in the neutralization of such reactive oxygen species (ROS) as superoxide anion radicals, free hydroxyl radicals, and hydrogen peroxide (H_2_O_2_). It is also involved in interactions of hydroxyl and amine groups with metal ions resulting in metal chelation, adsorption, and ion exchange [27,32]. However, research results have indicated that chitosan can also induce the synthesis of H_2_O_2_ in plant cells as a signal molecule in defense reactions to stress and increase the activity of superoxide dismutase (SOD), peroxidases (POX), and catalase (CAT), i.e., enzymes involved in the direct neutralization of ROS [33]. As reported by Hawrylak-Nowak et al. [34], spraying plants with a chitosan lactate solution increased CAT and guaiacol peroxidase (GPOX) activity in *Ocimum basilicum* and ascorbate peroxidase (APX) in *Melissa officinalis*. Similarly, foliar application of chitosan nanoparticles increased the activity of CAT, APX, and glutathione reductase (GR) in *Catharanthus roseus* exposed to salt stress [35]. In turn, Quitadamo et al. [36] reported an important role of chitosan as a regulator of antioxidant responses to salinity in *Triticum durum*. It was observed that the use of this polymer neutralized the harmful effects of stress through a reduction in the content of superoxide radicals, H_2_O_2_, and malondialdehyde and via enhancement of CAT activity. In turn, the addition of chitosan as an elicitor in *Psammosilene tunicoides* hair root cultures was found to induce the production of nitric oxide and increase the activity of ROS-scavenging enzymes [37]. A similar effect was observed in *Curcuma longa* [38], where chitosan contributed to an increase in the activity of POX and polyphenol oxidase (PPO). Additionally, the results of research on the induction of oxidative responses by salicylic acid, chitosan, and exogenous H_2_O_2_ in *Capsicum annuum* plants demonstrated that, in comparison with other substances, the foliar application of chitosan induced the lowest H_2_O_2_ accumulation in the leaves of this species [39]. The antioxidant activity of chitosan is also one of the criteria of its suitability to be used for enhancement of the storage stability of raw materials, which is described in detail in the next section. For example, in a study conducted by Wang and Gao [40], treatment of strawberries with chitosan induced a number of defense reactions; i.e., it increased the activity of CAT, GPOX, glutathione peroxidase (GSH-POX), dehydroascorbate reductase (DHAR), and monodehydroascorbate reductase (MDHAR).

The biological activity of chitosan and the viscosity of its solutions, and thus the effectiveness of its use, are determined by a number of different factors, e.g., the origin, type of modifications, polymerization and deacetylation degrees, pH, positive charge, concentration, solubility, and chelation capacity [26,41]. Recent studies also indicate the importance of the pattern of chitosan acetylation, which may be an important molecular carrier of information [42].

The effect of chitosan depends on the plant species, developmental stage, and physiological condition. In turn, the antimicrobial activity of this polymer depends on the type of microorganisms. However, molecular weight is the most important parameter with a large impact on the level of biological activity of chitosan [26,33]. For example, it was observed in an experiment carried out by Kulikov et al. [43] that a decrease in the molecular weight of chitosan was accompanied by an increase in its inhibitory activity against mosaic virus infection in *Phaseolus vulgaris*. Animal-derived chitosan is a high-molecular-weight polymer. Moreover, it exhibits antimicrobial activity only in an acidic environment, in which it is soluble. Therefore, it is important to develop technologies for modification and adaptation of chitosan in order to elicit adequately targeted biological activity of this compound [9,44].

## 3. Application of Chitosan as a Biostimulant in Cultivation of Plants

Plant production is one of the most important elements of agriculture and the economy, which require continuous progress. The introduction of various novel technologies has contributed to the rapid increase in agricultural performance, but the search for ecological solutions increasing the efficiency of plant production has currently become essential [33]. Pro-ecological activities in this area are also enforced by the latest changes in agricultural policies, such as the European Green Deal, with one of the strategies aimed at a reduction in the use of plant protection products and the promotion of organic farming [45]. Hence, the interest in the use of natural-origin substances as stimulants of plant growth and development, the so-called biostimulants, has increased in recent years. The concept of biostimulants was proposed at the end of the 20th century but has not been clearly defined to date. Moreover, there are no adequate legal regulations for the systematization of available preparations or registration of new agents. However, the term “plant biostimulant” is assumed to denote any substance or formulation that is not a plant constituent, fertilizer, or pesticide but contains natural compounds (single or mixtures) or microorganisms. It is intended to be applied onto the whole plant, a part of a plant, or the rhizosphere in order to intensify natural physiological processes, increase plant resistance to stress, enhance the utilization of minerals, and improve the size and quality of crop yields [46,47,48].

Given its biological activity and film-forming properties, chitosan is widely used in treatments improving the propagation material, such as coating or encapsulation. It is most often used as an independent bioactive compound or a binding substance in combination with other agents stimulating plant growth and development and ensuring protection against pathogens. It is believed that coating the propagation material with a solution of this polymer mainly ensures adequate water and gas permeability, thus stimulating germination and development of seedlings [25,49]. It is also suggested that chitosan can activate hydrolytic enzymes required for the degradation and mobilization of storage substances, such as starch and protein [50]. A study conducted by Ruan and Xue [51] showed that coating *Oryza sativa* seeds with chitosan accelerated their germination and increased the tolerance of these plants to salt stress. In turn, experiments carried out on tubers of freesia [52] showed that the application of a 0.2% chitosan solution increased the biomass and the number of progeny tubers in this species. Subsequent studies [53] demonstrated a positive effect of coating *Ornithogalum saundersiae* bulbs with a 0.5% aqueous solution of oligochitosan with 5000 or 100,000 g × mol^−1^ molecular weight on plant yields and most of the analyzed biometric and physiological indicators. It was also found that different doses of chitosan stimulated seed germination and had a positive effect on the fresh weight of cucumber roots and shoots [54]. Similar results were reported by Zeng et al. [55]; i.e., treatment with a chitosan solution stimulated germination and increased yields in soybean.

Current literature reports indicate a high effectiveness of foliar or soil application of chitosan in the stimulation of plant growth. This may be related to its stimulating effect on the uptake of water and essential minerals and its impact on the osmotic pressure in cells [41]. It was reported that a solution of this polymer sprayed on strawberry plants exerted a beneficial effect on plant growth and fruit yield [56]. This finding was confirmed in a study conducted by Rahman et al. [57], where foliar treatment of strawberry with chitosan had a positive effect not only on the growth and yield of fruits but also on their chemical composition. Furthermore, the results reported by Poterańska et al. [58] indicated that foliar application of chitosan-containing agents had a positive effect on the weight of haskap berries. In turn, *Eustoma grandiflorum* plants grown in soil with the addition of chitosan flowered much earlier and produced a larger number and greater weight of flowers [59]. Equally positive results were reported by Chookhongkha et al. [60], who additionally indicated a variable effectiveness of chitosan depending on its molecular weight. It was found that 1% high-molecular-weight chitosan applied to soil had a beneficial effect on fruit and seed yields in *Capsicum annuum*.

The literature provides information on the potential use of chitosan in the fertilizer industry as an ingredient for the production of sustained (SRF) or controlled release (CRF) fertilizers. The interest in this polymer has mainly been aroused by its beneficial effect on soil and plants and some physicochemical features. Given its film-forming properties as well as high biocompatibility and biodegradability, chitosan can be used as a coating material regulating the rate of release of minerals into the soil solution; additionally, it can be used to solve the problem with the disposal of residues of coatings produced from non-biodegradable polymers. In the agrochemical industry, chitosan nanoparticles are used both to optimize the activity and efficiency of various types of formulations and to reduce their toxicity to the environment [23,61,62]. An example of such an application was shown in an experiment carried out by Abdel-Aziz et al. [63], in which the foliar application of a nanochitosan-NPK fertilizer contributed to an increase in wheat growth and yields compared to plants treated with traditional forms of nitrogen, phosphorus, and potassium.

## 4. Chitosan as a Plant Protection Agent

The presence of harmful organisms poses a serious threat to agriculture and many industries worldwide. According to data published in 2019 by the Food and Agriculture Organisation of the United Nations (FAO), agrophages cause approximately 20–40% of losses of the world’s crops annually [64]. The use of chemical plant protection products raises increasing concerns related to their negative impact on the environment despite their effectiveness. Given the growing ecological and consumer awareness and changes in the agricultural sector, the search for natural and safe methods for the protection of crops against pathogens and pests has become relevant. Chitosan is commonly described in the literature as a stimulant of plant resistance inducing natural defense mechanisms, which may reduce the amounts of synthetic plant protection products used in cultivation [33]. With its film-forming properties, this polymer can also act as a physical barrier limiting the contact between plants and pathogenic microorganisms [65]. Moreover, due to their potent antimicrobial activity, chitosan-based products can be used separately or in combination with other agents as biocides applied as hydrogels for coating tubers, fruits, and seeds; as a solution for soil or foliar application during or after vegetation; or as medium supplements in hydroponic or tissue cultures [33]. Numerous studies have been carried out to assess the potential use of chitosan as a plant health-promoting agent. It was shown that soaking freesia tubers in a 0.2% solution of chitosan with different molecular weights (in the range of 2000–970,000 g × mol^−1^) exerted a positive effect on their health [52]. In turn, the treatment of cucumber seeds with chitosan increased the resistance of seedlings to *Phytophthora capsici* in a concentration-dependent manner (0, 125, 250, and 500 ppm). The highest concentration ensured complete resistance of young cucumber plants to blight caused by the pathogen [54]. Treatment of soybeans with a chitosan solution was reported to reduce the presence of herbivorous insects [55]. Moreover, chitosan was found to induce an effective reduction in the viability of *Phomopsis viticola* spores [66]. Given its properties, it is also possible to use this polymer for the protection of herbal plants against pathogens. Szczeponek et al. [67] demonstrated the efficacy of a chitosan-based formulation against fungal species that infest lemon balm and peppermint most frequently. Chitosan can also be used to control pathogenic soil nematodes and increase plant resistance to these pests [41].

Chitosan is also one of the few active substances contained in formulations and fungicides approved for use in forestry for the protection of forest nurseries against pathogenic diseases [68]. As shown in an experiment carried out by Aleksandrowicz-Trzcińska et al. [69], foliar application of a chitosan-containing formulation increased the resistance of Scots pine to fungal infections. Other results of laboratory studies indicated that chitosan exerted a negative effect on growth and caused changes in the morphological and structural structure of *Cylindrocladium floridanum*, *Cylindrocarpon destructans*, *Fusarium acuminatum*, and *Fusarium oxysporum* fungi responsible for root rot in forest nurseries [70]. Moreover, there are a number of chitosan-based agents on the market for coating injuries and wounds in trees and shrubs.

## 5. Application of Chitosan in Storage

The main goal of storage is to reduce food losses by ensuring the longest possible shelf life, good quality of stored products, and the best possible protection of product stability before further distribution. An important role in this process is mainly played by the selection of optimal conditions and storage methods as well as the monitoring of such product quality parameters as the color, weight, firmness, content of bioactive compounds, and rate of ethylene and carbon dioxide production. However, in the case of low-processed raw materials, it is often necessary to employ various types of preservation methods. One of the options to preserve the quality of stored raw materials consists in a reduction in respiration and transpiration through the use of natural edible coatings. The potential of using chitosan in storage is associated with its physicochemical properties and biological activity. Given its natural origin, ability to form semipermeable coatings, and high antioxidant and antimicrobial activity, this polymer facilitates the maintenance and optimization of the postharvest stability of raw materials and food products and influences their chemical composition. Additionally, chitosan delays fruit ripening through the limitation of ethylene and carbon dioxide release. Therefore, it can be widely used in the production of biodegradable films, food packaging, fibers, gels, and films with high strength and flexibility. It can also be used as nanoparticles. Moreover, this polymer can be applied directly to fruits and vegetables as a single ingredient or in combination with other substances in order to create edible protective coatings with bacteriostatic and fungistatic properties [28,44,71]. Modifications achieved by combining chitosan with various types of substances offer many possibilities for the production of edible coatings and food packaging with required properties. For instance, conjugates of chitosan with phenolic (gallic and caffeic) acids produced durable films with adequate properties to serve as a barrier against water vapor and oxygen. They exhibited stronger antioxidant and antimicrobial activity than films based on traditional chitosan [72].

Numerous reports have demonstrated the suitability of chitosan for storage purposes, as its properties help to enhance the stability and quality of stored products. One of the examples is the research on the effect of chitosan coatings on the shelf life and quality of plum fruit. The results of the experiment showed that the use of coatings containing 2% chitosan had a significant effect on the maintenance of the color, firmness, and weight of fruit stored at low temperature [73]. Similar results were obtained in a study of guava fruit stored at low temperature; i.e., the use of 2% chitosan coatings exerted a positive effect on the quality, firmness, and weight of fruit [74]. It was also observed that chitosan coatings increased the antioxidant properties of apricot fruits and contributed to the longer maintenance of a high total content of phenolic compounds [75]. Moreover, another experiment showed that chitosan coatings increased the storage stability of longan fruits and had a positive effect on their quality, color, and weight during storage [76]. A similar effect was reported in a study of *Actinidia melanandra* fruits, whose storage stability increased after the application of gel coatings containing this polymer [77]. In an experiment conducted by Zhu et al. [78], the application of chitosan coatings delayed the maturation and degradation of mangoes.

A study conducted by Tayel et al. [79] demonstrated that coating lemon fruit with chitosan inhibited the occurrence of *Penicillium* fungi. Moreover, the application of a chitosan-based formulation during the potato vegetation period was found to exert a limiting effect on the presence of *Fusarium* spp. and *P. carotovorum* subsp. *carotovorum* causing dry and wet tuber rot during storage [80]. A similar protective effect against the presence of the bacterial pathogen *Acidovorax citrulli*, which causes fruit blotch, was achieved in the case of watermelon seedlings [81]. In turn, as reported by He et al. [82], the use of a chitosan spray before harvesting strawberries had a beneficial effect on the quality and shelf life of the fruit through maintenance of the content of sugars; vitamin C; and numerous secondary metabolites, e.g., total phenolic compounds, flavonoids, and anthocyanins. Furthermore, the use of different concentrations of chitosan with different molecular weights was shown to increase the resistance of tomato fruit to grey mold caused by *Botrytis cinerea*. This was associated with both the direct antifungal activity of chitosan and the induction of a biochemical defense response in the fruit, which resulted in increased accumulation of phenolic compounds and enhanced activity of PPO [83].

## 6. Chitosan and its Derivatives as Biotic Elicitors

The potential for application of chitosan and its derivatives in the elicitation process is related to the biological activity of this compound, which primarily involves the ability to stimulate natural plant defense mechanisms and increase plant resistance to stress. This is associated with various types of physiological and biochemical changes, such as oxidative stress; accumulation of H_2_O_2_; synthesis of secondary metabolites (polyphenolic compounds, phytoalexins, flavonoids, alkaloids), enzymes (chitinase, glucanase, protease), and growth inhibitors (abscisic acid, jasmonic acid, salicylic acid); and accumulation of lignin and callose. The effect of chitosan on plants is reflected in changes in the chromatin structure, the inhibition of H^+^-ATPase activity in the cell membrane, the activation of MAP kinases, and an increase in the cytosolic Ca^2+^ concentration [23,44,84]. Elicitation is aimed at stimulation of the biosynthesis of secondary metabolites present in plants or induction of the formation of new substances. It can solve problems related to the insufficient amount of bioactive compounds produced by plants, which does not cover the current needs, and bring benefits by providing high-quality raw materials with increased content of health-enhancing compounds [85]. The effectiveness of chitosan in elicitation largely depends on its solubility; therefore, chitosan salts, such as lactate or acetate, are the most commonly used derivatives of this polymer [21,34].

Numerous research publications report the effectiveness of chitosan and its derivatives in the elicitation process. Studies on *Curcuma longa* [86] indicated that the foliar application of a 0.1% chitosan solution triggered defense responses in the plants and had a positive effect on both plant growth and accumulation of curcumin in their rhizomes. A stimulating effect of 0.1% and 0.2% chitosan solutions was also reported in a study of *Stevia rebaudiana*. The application of chitosan increased the biomass and concentration of phenolic compounds and rebaudioside A [87]. A field experiment conducted on *Origanum vulgare* ssp. *hirtum* demonstrated that the use of different concentrations of chitosan oligosaccharides (50, 200, 500, or 1000 ppm) exerted a beneficial effect on plant growth and the content of polyphenolic compounds [88].

The study conducted by Gerami et al. [89] suggested the possibility of the use of chitosan as an elicitor increasing *S*. *rebaudiana* tolerance to salinity and reducing its phytotoxic effects on these plants. Similar results were obtained in an experiment conducted by Safikhan et al. [90], in which chitosan mitigated the harmful effects of salt stress, stimulated growth, and had a positive effect on the physiological parameters of *Silybum marianum*. Furthermore, a study conducted in water deficit conditions showed that chitosan treatment of *Salvia officinalis* reduced the negative impact of drought stress. It also had a positive effect on the quantity and quality of essential oil, the total content of phenolic compounds and flavonoids, and the antioxidant properties of sage extracts [91]. A stimulating effect of a chitosan suspension and a chitosan solution in 1% acetic acid on the production of flavonoids in *Ononis arvensis* in in vitro conditions was demonstrated as well [92]. A chitosan solution in acetic acid used as an elicitor in *Mentha piperita* suspension cultures produced a significant increase in the accumulation of menthol [93]. In turn, the treatment of *M. piperita* with chitosan in greenhouse conditions increased the total content of phenolic compounds and flavonoids and enhanced the antioxidant activity of the extracts [94]. Chitosan also stimulated the production of triterpenoid saponins in *Psammosilene tunicoides* hair root cultures [37]. Moreover, the polymer was found to have a beneficial effect on biomass accumulation in cell cultures of three basil species: *Ocimum basilicum*, *O. sanctum*, and *O. gratissimum* [95]. Other studies carried out on *O. basilicum* indicated the effectiveness of chitosan elicitation in the stimulation of biomass growth, intensified accumulation of total phenolic and terpene compounds, elevation of rosmarinic acid and eugenol concentrations, and enhancement of antioxidant activity [96]. The foliar application of chitosan lactate stimulated the biosynthesis of rosmarinic acid, anthocyanins, and phenolic compounds in *O. basilicum* and *M. officinalis* raw materials [34].

The effects of the application of chitosan and its derivatives on the accumulation of secondary metabolites in selected plant species are shown in Table 1.

## 7. Prospects for New Applications of Chitosan

Due to the wide availability and a number of its beneficial properties, an increase in the number of potential applications of chitosan and its derivatives has been observed in recent years. Nevertheless, despite its numerous advantages, the practical commercial-scale use of this polymer is still limited by some of its physicochemical features and is dependent on progress in the development of optimal methods for chemical modification and adaptation of the polymer for specific applications. To date, the greatest success in this regard has been achieved in nanotechnology, which is regarded as one of the five most groundbreaking fields of science and technology of the last century [6,104,105]. The achievements of this discipline facilitate a wide use of chitosan nanomaterials in various types of biomedical, cosmetic, food, ecological, and agrotechnical innovations [13,16]. The application of nanoparticles facilitates more effective utilization of the biological properties of chitosan.

One of the most important prospects for the use of chitosan in nanotechnology is the concept of sustainability of the agrochemical industry through the production of the so-called agronanochemicals. It mainly involves the preparation of chitosan nanoparticles and chitosan nanocomposites or encapsulation of active substances in chitosan-based nanocarriers. The preparations primarily exhibit high efficiency and bioavailability; hence, they can constitute a more economical environmentally friendly alternative limiting the amounts of currently available chemicals [17,104,106,107].

Chitosan may exert a positive effect on plant photosynthetic efficiency, ability to biosynthesize and accumulate chlorophyll, and nutrient uptake [27,106,108,109]. The available research results demonstrate that chitosan nanoparticles have a greater impact on the macronutrient uptake efficiency than chitosan oligomers. Experiments carried out in greenhouse conditions showed that chitosan nanoparticles contributed to an increase in photosynthetic efficiency; chlorophyll content; and nitrogen, phosphorus, and potassium absorption in *Coffea canephora* [110]. Similar results were reported by Sathiyabama and Manikandan [111], who found that the foliar application of nanochitosan stimulated growth and increased the mineral content in *Eleusine coracana*.

Various research results also indicate the possibility of using chitosan to increase plant tolerance to abiotic and biotic stress factors and mitigate their phytotoxic effects [35,107,109]. Many reports show the effectiveness of chitosan in increasing plant resistance to salinity, drought, or low-temperature stress [112,113]. It was shown that spraying with a solution containing 1% chitosan nanoparticles alleviated the effects of salt stress through the activation of defense mechanisms in *Catharanthus roseus* [35,109]. The foliar application of a solution containing chitosan nanoparticles (400 mg L^−1^) to bananas enhanced their tolerance to low temperature and improved biometric parameters via a reduction in the oxidative stress intensity [113]. In turn, Kocięcka and Liberacki [112] reported the effectiveness of chitosan, its derivatives, and nanoparticles in the stimulation of the resistance of cereals to such abiotic stress factors as low temperature, drought, and salinity and in the enhancement of the health of plants and improvement of their quality.

This polymer can also be used in combination with other compounds not only to extend the spectrum of its activity but also to produce materials with more favorable mechanical, chemical, thermal, and barrier properties [17,107,114]. The use of chitosan in the production of nanocomposites may also increase the effectiveness of other chemical compounds or elements, e.g., zinc, iron, copper, and silver [107,114]. In addition to the stimulation of the efficiency of agrochemicals, these nanocomposites enhance the biosynthesis of secondary metabolites and may accelerate plant regeneration. An example of this type of use is the application of zinc encapsulated chitosan nanoparticles, which had a beneficial effect on the growth, health, and quality of maize kernels [115]. In turn, studies of *Capsicum annuum* tissue cultures showed that a composite of zinc oxide and chitosan nanoparticles contributed to a significant increase in its elicitation efficiency [116].

Chitosan nanoparticles can reduce the amount and frequency of application of plant protection products, ameliorate their negative effects, and help to monitor and remove their excess from soils and waters [23,117]. Due to its cationic nature and high affinity to metals and dyes, chitosan is also one of the preferred natural and relatively cheap adsorbents replacing the currently used active carbon, with similar sorption properties in water engineering [2,10,105,118]. In water remediation, similar to other applications, chitosan can be used alone or in combination with other substances, e.g., activated carbon or silicon dioxide. Nanocomposites made of chitosan and activated carbon, whose combination ensures a significant increase in their adsorption capacity, are one example [118,119].

**Figure 2:**
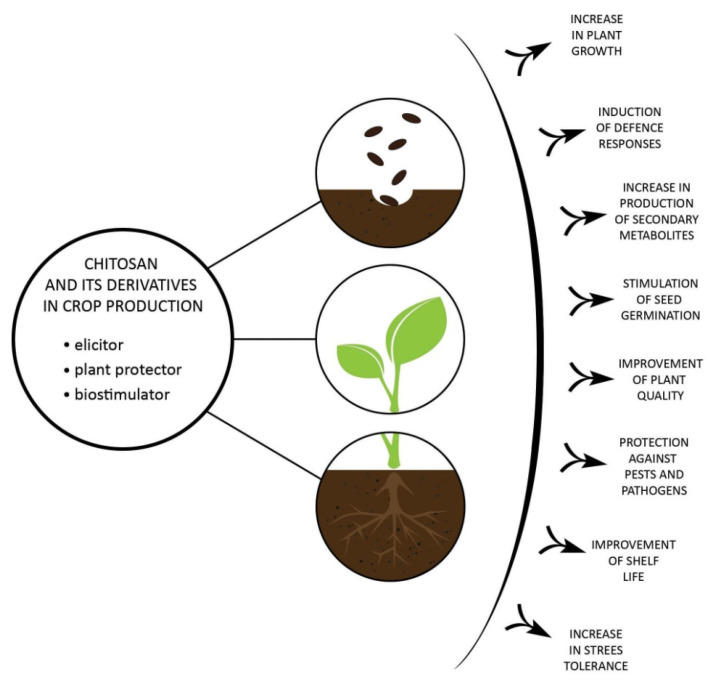
Application of chitosan and its derivatives in plant production.

The main applications of chitosan and its derivatives in plant production are summarized in Figure 2.

## 8. Conclusions

Chitosan is an easily available environmentally friendly biopolymer with numerous favorable biological properties; hence, it has many applications in various fields. Additionally, it can be a potential solution to various ecological problems, especially in the production of plants by increasing their internal potential. Biodegradable and biocompatible chitosan and chitosan-based nanomaterials are becoming essential in agriculture due to their unique properties, such as biostimulating, eliciting, and antimicrobial activity and stimulation of plant growth and tolerance to environmental stresses. However, the use of other effective sources of chitosan, the methods for extraction and optimization of its physicochemical properties, and the practical implementation of laboratory results should be investigated comprehensively. Moreover, detailed studies on the potential toxicity of chitosan-based nanomaterials and the ecological consequences of their large-scale use are required. At present, these substances are not being widely used in agriculture, as the mechanisms of their biological activity in plants and action against pathogenic microorganisms have not been fully elucidated to date.

## Figures and Tables

**Table 1:** Effects of application of chitosan and its derivatives on the level of secondary metabolites in selected plant species.

Plant Species	Plant Growth Conditions	Chitosan Form	Dose, Method, and Number of Chitosan Applications	Effect of Chitosan on the Level of Secondary Metabolites	Reference
*Artemisia annua*	laboratory conditions; hairy root cultures	chitosan	50, 100, or 150 mg L^−1^ of chitosan added to hairy root cultures	increased artemisinin production	Putalun et al. [97]
*Curcuma longa*	field conditions	chitosan	0.1% chitosan; foliar application; seven treatments	increased curcumin content	Sathiyabama et al. [38]
*Dracocephalum kotschyi*	glasshouse; mixture of peat, sandy soil, and perlite substrate	chitosan	100 or 400 mg L^−1^ of chitosan; triple foliar application	enhanced biosynthesis of total phenolic and flavonoid compounds, including rosmarinic acid and apigenin	Kahromi and Khara [98]
*Catharanthus roseus*	greenhouse; sandy soil	chitosan nanoparticles	1% chitosan nanoparticles; single foliar application in salinity stress conditions	increased alkaloid accumulation	Hassan et al. [35]
*Fragaria × annanasa*	field conditions	chitosan	125, 250, 500, or 1000 ppm chitosan; foliar application; six treatments	increased amount of phenolic compounds, carotenoids, flavonoids, and anthocyanins in strawberry fruits	Rahman et al. [57]
*Ginkgo biloba*	laboratory conditions; callus cultures	chitosan	25, 50, 100, or 200 mg L^−1^ of chitosan added to MS medium	enhanced production of total flavonoids and total phenolic compounds	Elateeq et al. [99]
*Iberis amara*	laboratory conditions; cell suspension cultures	chitosan	50, 100, or 200 mg L^−1^ of chitosan	enhanced total phenolic compounds, flavonoid, flavonol, and anthocyanin contents	Taghizadeh et al. [100]
*Isatis tinctoria*	laboratory conditions; hairy root cultures	chitosan	50, 100, 150, 200, or 400 mg L^−1^ of chitosan; hairy root cultures elicited for 6–96 h	increased total flavonoid accumulation	Jiao et al. [101]
*Melissa officinalis*	phytotron room; soil substrate	chitosan lactate	100 or 500 mg L^−1^ of chitosan lactate; single foliar application	increased accumulation of rosmarinic acid, anthocyanins, and total phenolic compounds	Hawrylak-Nowak et al. [34]
*Mentha piperita*	greenhouse; soil phosphate	chitosan	50 or 100 µM chitosan; single foliar application	increased content of phenolic and flavonoid compounds	Salimgandomi and Shabrangi [94]
*Ocimum basilicum*	greenhouse; potting substrate irrigated with a fertilizer solution	chitosan	0.01%, 0.05%, 0.1%, 0.5% or 1% chitosan; seed soaking (30 min.)	increased content of total phenolic and terpenic compounds (rosmarinic acid, eugenol)	Kim et al. [96]
*Origanum vulgare ssp. hirtum*	field conditions	chitosan oligosaccharides	50, 200, 500, or 1000 ppm chitosan oligosaccharides; single foliar application	increased accumulation of total flavonoids and total polyphenolic compounds	Yin et al. [88]
*Psammosilene tunicoides*	laboratory conditions; hairy root cultures	chitosan	200 mg L^−1^ of chitosan; hairy roots elicited by chitosan for nine days	enhanced accumulation of total saponins, increased content of quillaic acid, gypsogenin, and gypsogenin-3-O-β-D-glucuronopyranoside	Qui et al. [37]
*Salvia officinalis*	field conditions	chitosan	0.25 or 0.50 g L^−1^ of chitosan; single foliar application in reduced irrigation conditions	increased amount of total phenolic and flavonoid content; enhanced production of α- and β-pinene, limonene, α- and β-thujone, camphor, and 1,8-cineole in the essential oil	Vosoughi et al. [91]
*Satureja isophylla*	greenhouse; sandy soil	chitosan	0.2 or 0.4 g L^−1^ of chitosan; foliar application	increased amount of essential oil; increased concentrations of essential oil constituents (carvacrol, β-bisabolene)	Salehi et al. [102]
*Stevia rebaudiana*	greenhouse; perlite and peat substrate	chitosan	0.2, 0.4, or 0.6 g L^−1^ of chitosan; double foliar application in salinity stress conditions	increased content of steviol glycosides: stevioside and rebaudioside A	Gerami et al. [89]
*Sylibum marianum*	laboratory conditions; cell suspension cultures	chitosan	0.5, 1, 2.5, 5, 10, 25, or 50 mg L^−1^ of chitosan in MS medium	enhanced accumulation of total flavonoids, total phenolic compounds, and silymarin	Shah et al. [103]

## Data Availability

Not applicable.
